# CT slice index and thickness: Impact on organ contouring in radiation treatment planning for prostate cancer

**DOI:** 10.1120/jacmp.v4i4.2511

**Published:** 2003-09-01

**Authors:** E. Berthelet, M. Liu, P. Truong, P. Czaykowski, N. Kalach, C. Yu, K. Patterson, T. Currie, S. Kristensen, W. Kwan, V. Moravan

**Affiliations:** ^1^ Department of Radiation Oncology BCCA‐Vancouver Island Centre 2410 Lee Avenue Victoria BC V8R 6V5 Canada; ^2^ Department of Radiation Oncology BCCA‐Fraser Valley Centre 13750 96th Avenue Surrey BC V3V 1Z2 Canada; ^3^ Division of Hematology/Oncology, Department of Medical Oncology University of Manitoba, CancerCare Manitoba 675 McDermot Avenue Winnipeg MB R3E 0V9 Canada; ^4^ Population and Preventative Oncology BCCA‐Vancouver Centre 600 W 10th Avenue Vancouver BO V5Z 4E6 Canada

**Keywords:** CT slice thickness, CT slice index, prostate cancer, partial volume effect

## Abstract

*Objective:* To assess the impact of CT slice index and thickness (3 mm versus 5 mm) on (i) prostate volume, dimensions, and isocenter coordinates, (ii) bladder and rectal volumes, and (iii) DRR quality, in the treatment of prostate cancer. *Methods:* 16 patients with prostate cancer underwent two planning CT‐scans using 3 and 5 mm slice index/thickness. Prostate, bladder, and rectum were outlined on all scans. Prostate isocenter coordinates, maximum dimensions, and volumes were compared along with bladder and rectal volumes. Bladder volumes and maximum diameters were further investigated using a second observer. A comparative analysis of DRR quality was conducted as well as a dosimetric analysis using DVH. *Results:* The differences in measurements of prostate volume, isocenter coordinates and maximum dimensions between the 3 and 5 mm scans, were small and not statistically significant. Similar finding was seen for rectal volume. However, bladder volume was always larger on the 3 mm scan (mean difference=27.9 cc;  SE=4.8 cc;  95% CI:  17.7−38.2 cc;  p<0.001) and the findings were reproduced with the second observer (mean difference=31.9 cc;  SE=4.7 cc;  95% CI:  21.9−41.9 cc;  p<0.001). The differences in volume are caused by a slight increase in (1) the measurement of the longitudinal dimensions on the 3 mm scans, and (2) the slice by slice measured bladder area on the 3 mm scans. The latter is due to partial volume effect. The 3 mm DRR were slightly better than the 5 mm DRR. The bladder DVH differed significantly in some patients. *Conclusion:* Bladder volume is significantly larger on the 3 mm scans. Differences in contoured areas may be accounted for, in part, by the partial volume effect.

PACS number(s): 87.57.–s, 87.53.–j

## INTRODUCTION

Several factors have been shown to influence the delineation of the prostate in the treatment planning process of external beam radiotherapy (EBRT) for prostate cancer. The use of contrast agents^1^ ^‐^ ^4^ or fiducial markers,^2^ ^,^ ^5^ for example, may help define the superior and/or inferior limit of the prostate, prostate volume itself, and/or its maximum dimensions. The interobserver variability inherent to organ volume definition can also impact on the delineation of the prostate and the placement of the isocenter coordinates.^4^ ^,^ ^6^ ^‐^ ^12^ The imaging modality selected (CT, US, or MRI), may also influence the measurement of certain parameters such as organ volume.^13^ ^,^ ^14^ Finally and not the least, prostate motion itself, either during the whole course of treatment,^5^ ^,^ ^15^ ^‐^ ^18^ or within a single fraction,^19^ ^‐^ ^22^ may lead to variations in isocenter position and hence field placement or margin determination.

CT remains the most widely used imaging modality in the treatment planning of EBRT for prostate cancer. Although it has been suggested that the choice of a particular CT slice thickness or CT slice index (spacing) may influence organ delineation and volume determination,^9^ this phenomenon has not been extensively investigated before.

In order to assess its impact, we elected to study the effect of 3 mm versus 5 mm CT slice index/thickness on prostate, bladder, and rectum volumes as well as prostate dimensions and isocenter coordinates, as they apply to the treatment planning of prostate cancer with EBRT. A comparative analysis of digitally reconstructed radiographs (DRR) quality using five blinded observers was conducted. Finally, a dosimetric analysis using dose volume histograms (DVH) was conducted to assess the potential clinical relevance of the findings.

## MATERIALS AND METHODS

Sixteen patients with organ confined (T1 and T2), histologically confirmed adenocarcinoma of the prostate were selected for the study. Consent was obtained from all patients. Each underwent two CT scans for planning purposes. The studies were acquired on a Picker 2000–AcQSIM unit at 5 mm/5 mm and 3 mm/3 mm index/thickness respectively while all other parameters of the scanning protocol remained the same. Patients were instructed to have a full bladder for the CTs. Prostate, bladder, and rectum were outlined on all scans using a single observer. Organ volumes were recorded on both scans for all 16 patients. The algorithm used to determine organ volume is a simplified version of the sum‐of‐polygons technique. It is calculated as the sum of the organ area on each slice multiplied by the slices separation. In addition, the maximum dimensions in the anterior‐posterior (AP), lateral (Lat), and longitudinal (Long) dimensions as well as the isocenter coordinates in the Lat (*X*), AP (*Y*), and Long (*Z*) directions were recorded for the prostate only. Measurements between scans were compared using the Paired Samples T‐Test. Bladder volume and maximum dimensions were further investigated by asking a second observer to outline the bladders on all CT's. Statistical analysis was performed using the Paired Samples T‐Test and a two level (slice index/thickness and rater) Repeated Measures ANOVA.

A comparative analysis of DRR quality was also conducted. In the clinical setting, DRR quality was defined as its usefulness in the accomplishment of a specific task: the verification of portal images taken at the beginning of the treatment. Both DRRs were obtained using the same window and level. Five blinded observers were shown anterior (AP) and right lateral (Rt Lat) projections of the treatment fields obtained from both CTs (5 and 3 mm) for all 16 patients. They were asked to score one of the DRRs (3 or 5 mm) on a scale of 1 to 5 (much better, better, comparable, worse, much worse) in comparison to its counterpart. The binomial probability test was used to assess the statistical significance of the distribution of ratings and an analysis of the variance components was conducted.

## RESULTS

*1. Organ volume, prostate dimesions and isocenter coordinates*. The results of the initial analysis are presented in Table I. In order to correct for multiple statistical testing, a value of p≤0.005 was preselected as the level of statistical significance. This stems from the fact that nine different parameters were analyzed. Therefore, dividing the commonly used *p* value ≤0.05 by 9 (we chose 10 for simplicity: p≤0.05/10; p≤0.005) would greatly reduce the chance of reaching statistical significance by chance and strengthen our conclusions. From Table I, it becomes clear that the bladder volumes are larger on the 3 mm scans. The average difference is 27.93 cc (SD=19.21 cc). Although the longitudinal dimension of the prostate was generally larger on the 3 mm scans, this did not reach statistical significance using the criteria cited above (p=0.03). Interestingly, no trend was observed in terms of prostate volume. The mean difference between the 3 and 5 mm scans was only −0.19 cc and it did not reach statistical significance (p=0.855).

**Table I:** Mean differences of measurements (3–5 mm). X, Y, and Z represent the prostate isocenter coordinates while Lat, AP, and Long represent the prostate maximum dimensions. X, +/− ◊ left/right; Y, +/− ◊ post/ant; Z, +/− ◊ sup/inf

Measurement	Mean	SD	*P* value
*X* (mm)	−0.01	1.21	0.984
*Y* (mm)	0.88	2.83	0.234
*Z* (mm)	1.13	2.05	0.044
Lat (mm)	0.99	2.58	0.147
AP (mm)	0.34	2.90	0.643
Long (mm)	2.18	3.72	0.033
Prostate Vol. (cc)	–0.19	4.13	0.855
Rectum Vol. (cc)	0.15	17.76	0.972
Bladder Vol. (cc)	**27.93**	**19.21**	**0.001**

**Figure 1:**
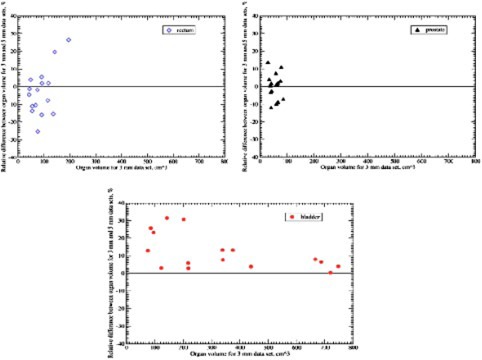
(Color) Relative volume difference as a function of the organ volume measured on the 3 mm scan for the rectum, bladder, and prostate.

Figure 1 depicts similar findings for organ volumes in the 16 patients. The relative volume differences (3–5 mm %), are plotted as a function of the 3 mm organ volumes. Although prostate and rectal volumes are randomly distributed above and below the zero line, all the bladder volumes are distributed above, indicating that the bladder volume on the 3 mm scans was larger than the one on the 5 mm scans, for all 16 patients.

*2. Bladder volume*. Since the bladder volume was larger on the 3 mm scans and because this finding was systematically observed in all 16 patients, we asked a second observer to re‐outline all the bladders on both series of scans. This second observer was blinded to the outlines of the first observer. The comparative results of both observers are presented in Table II. Once again, it becomes clear that bladder volume, as delineated by observer 2, was also consistently larger on the 3 mm scan (p≤0.001). Interestingly, a trend was also seen between observers. The bladder volumes outlined by observer 2 were consistently larger than the ones outlined by observer 1, and this was noted with both the 3 and 5 mm scans (interobserver variability). The differences, however, were small. Analysis of variance components revealed that the difference in volumes was related to the choice of index/thickness (p<0.001), rather than to the observers themselves or any observer‐index/thickness interaction.

**Table II:** Comparison between both observers for bladder volume using the Paired Sample T‐test.

Bladder volume mean difference 3–5 mm		SE	95% CI	*P* value
Observer 1	27.9 cc	4.8 cc	17.7–38.2 cc	<0.001
Observer 2	31.9 cc	4.7 cc	21.9–41.9 cc	<0.001

*3. Bladder dimensions*. Comparative analysis of bladder dimensions in the AP, Lat, and Long was also conducted. For observer 1, the maximal extent of the bladder in the longitudinal axis, was larger on the 3 mm scan in 16/16 patients. The same finding was seen for observer 2 in 15/16 patients. The results indicate that the longitudinal diameter of the bladder was systematically slightly larger on the 3 mm scans compared to the 5 mm scans. This is not surprising considering that the 3 mm scans have better spatial resolution along the *Z* axis. However, this difference is too small to account for an interscan bladder volume difference as large as 30% in some cases.

**Figure 2:**
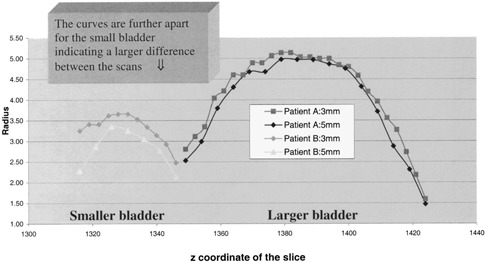
Impact of bladder size/shape on the interscan volume difference.

*4. Bladder contouring differences*. To investigate the impact of contouring differences on bladder volume, we selected two patients, one with large (29%) and one with small (8%) interscan bladder volume differences. The area of the bladder outlined on each slice of both scans was analyzed using the AcQSim VoxelQ measuring tool. We found that the measured area of the bladder was always larger on the 3 mm scans and that it was so on every CT slice. Furthermore, the differences in areas were larger for the patient who had a small bladder. The results are graphically represented in Fig. 2, where the effective radii of the bladder for 3 and 5 mm scans are shown for the two patients as a function of the CT slice *Z* position. Effective radius is defined as the radius of a circle of the same area as the bladder on a given CT slice. The organ volume in this case is calculated according to the simplified sum‐of‐polygons technique, where

V=[Σ (Area)]×(Thickness)

the sum of the contoured area on each slice multiplied by the slice thickness. Consequently, the interscan volume difference for 3 and 5 mm is related to the observed slice‐by‐slice difference in contoured area. The partial volume effect as a plausible reason for the observed area differences is discussed further below.

*5. DRR analysis*. Five mutually blinded observers compared DRR from the 3 and 5 mm scans. Two views, AP and Rt LAT, from 16 patients were presented in random order with respect to scan thickness/index. The observers were asked to rate the first of each 3–5 mm pair on a scale of 1–5 as described above. Out of a total of 160 comparisons, 6.9% (11) rated images from the 3 mm scans as “much better,” 58.1% (93) rated images from the 3 mm scans as “better,” 34.4% (55) rated the two images as “comparable,” and 0.06% (1) rated an image from the 3 mm scan as “much worse.” A binomial test of the 105 cases where a preference was expressed yielded a *p* value of <0.001 in favor of the 3 mm scans. Analysis of variance components showed that the difference in ratings was larger between observers than between patients or image incidences.

**Figure 3:**
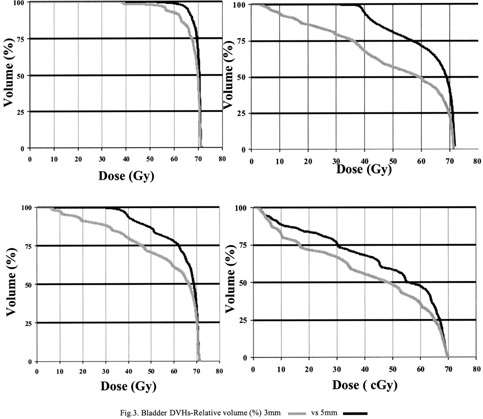
Bladder DVHs‐Relative volume (%) 3 mm 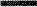 vs 5 mm 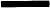.

*6. Dosimetric analysis*. As can be seen in Fig. 1, the relative difference in bladder volume between the scans, exceeded 20% in 4/16 patients (25%). The DVH analysis was restricted to those four patients. Figure 3 depicts the DVH data for those four patients in terms of relative volume. Since the bladders were larger on the 3 mm scans, a smaller percent of their overall volume received a given dose of EBRT (Fig. 3).

## DISCUSSION

With the development of three‐dimensional conformal radiotherapy and dose escalation, the precise delineation of the target volume has become an utmost priority. As discussed above, several factors may impact on the accuracy of target delineation. Prostate motion has long been recognized as an important limitation to dose escalation in the treatment of prostate cancer with EBRT. This phenomenon has been reported by various authors.^5^ ^,^ ^15^ ^‐^ ^23^ It has been suggested that the implantation of marker seeds directly into the prostate, may help circumvent this problem.^5^ ^,^ ^18^ ^,^ ^24^ The main advantage of this technique in our view, would be to allow corrections to the treatment or field set up prior to each fraction as suggested by Vigneault.^24^ The problem of seed displacement, however, may be a source of error in some cases; furthermore, it is an invasive procedure, not without its risks and complications.

The use of contrast agents has also been shown to improve the delineation of the target volume. Retrograde urethrography is still considered as one of the most accurate ways to determine the location of the prostatic apex.^13^ Rectal balloons have been used in some centers and shown to decrease the movement of the prostate.^25^ ^,^ ^26^

The choice of imaging modality has also been investigated extensively.^8^ ^,^ ^13^ ^,^ ^14^ It is a well known fact that CT tends to overestimate prostate volume when compared to transrectal ultrasound (TRUS)^13^ and MRI.^14^ MRI has been shown to decrease the variability associated with CT in delineating the apex of the prostate,^8^ potentially decreasing treatment toxicity.^14^ CT, however, remains a widely used modality for EBRT since most treatment planning systems depend on CT numbers for the dose calculation process.

Finally, there have been several reports on the interobserver variability inherent to the target delineation process or contouring.^6^ ^‐^ ^11^ This may in fact be the most important source of variation in some cases. Intraobserver variability has also been studied, although usually, its magnitude is less than that of interobserver variation.^8^ ^,^ ^9^

Another potential source of variation is the selection of CT slice thickness and index. Although this has been referred to by some authors,^9^ we could not find a thorough analysis of this phenomenon in the contemporary literature. Although we expected to see small differences between the 3 and 5 mm scans, our results for the bladder volume were somewhat surprising. Although the reason for this difference in bladder volumes may be multifactorial, we have identified the following three potential explanations which may, in some cases, be interrelated.

### A. Bladder filling effect

The protocol was designed to obtain the 5 mm scan first immediately followed by the 3 mm scan. We postulated that the timing of the scans may explain our findings, even though the scans were done only a few minutes apart from each other. When the 16 patients were reviewed, we found that in two cases, the 3 mm scan was obtained *before* the 5 mm scan. Despite this, the relative difference in bladder volume between the two scans were 4% and 5% for the first patient and 15% and 17% for the second patient as delineated by observers 1 and 2, respectively. In all cases the bladder volume was larger on the 3 mm scans, consequently, the bladder filling occurring during the interscan interval, could not solely explain the systematic volume difference we have observed. However, if a difference of a few minutes between scans contributed, at least in part, to our findings, one may wonder what the impact bladder filling may have during a radiotherapy fraction that lasts several minutes. Several recent reports have attempted to describe prostate motion during a radiotherapy fraction,^19^ ^‐^ ^22^ however, the changes of bladder volume and/or filling during the same interval are not well known. Although changes in bladder volume may have only minimal impact on the position of the prostate as suggested by some,^17^ this change, if it occurs in a significant manner, may impact the actual bladder dose and volume relationships which in turn could impact on toxicity. This dynamic phenomenon cannot be accounted for solely by reviewing DVHs which only provide us with a snapshot of the relationships between dose and volume of a given organ.

### B. Missing tissue effect

**Figure 4:**
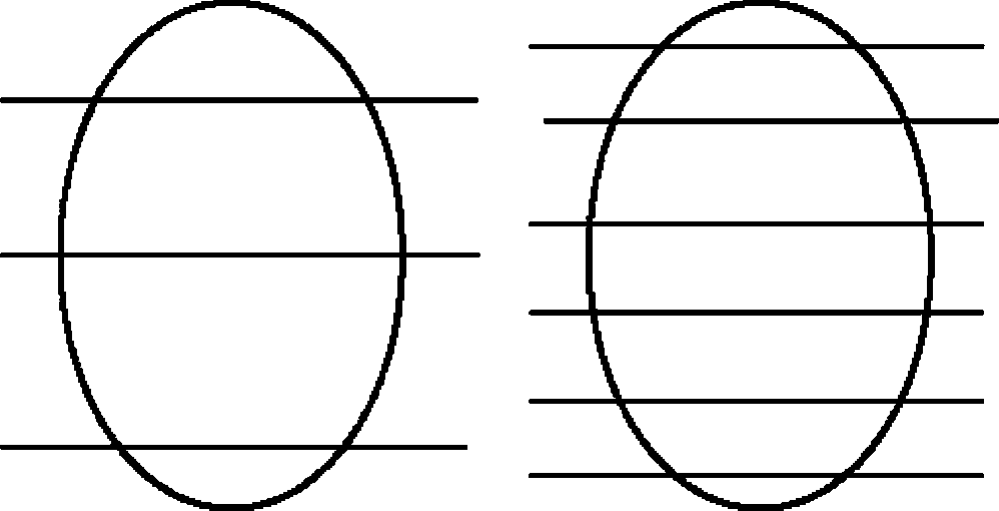
Missing tissue effect: The superior and inferior poles of the bladder may be missed if the index is too large.

As the index is decreased from 5 to 3 mm it becomes conceptually possible that the slices with the wider spacing (or larger index) may miss tissue at the cephalad and caudate end of the organ considered. This is illustrated in Fig. 4. Although this may be a contributing factor, it is unlikely that this phenomenon would be the sole explanation for the systematic finding described above. The smaller index may, at best, increase the probability that more tissue of a given organ will be captured.

### C. Partial volume effect

Partial volume effect is a well known CT image artifact.^27^ A CT number, in a given voxel of tissue, is generated based on the linear attenuation coefficient of the tissue present in that voxel. If two or more structures, with different densities, are present in a given voxel, the CT number of the voxel will be based on an average of those structures. The respective details of each individual structure may therefore be lost since an average CT number will be computed for each voxel.^28^ This effect is also known as partial volume averaging. Partial volume effect depends on the spatial resolution along the *Z* axis and hence, slice thickness, and affects the quality of the CT images and the ability to visualize fine structures.^29^ ^‐^ ^31^

**Figure 5:**
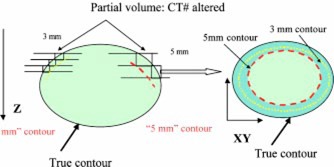
(Color) Left, partial volume effect on bladder contour along the *Z* axis; right, resulting blurred region as seen on a transverse CT slice along the *X* and *Y* axes.

The impact of this partial volume effect on organ contouring and delineation has not been studied before. Its influence on the capability of distinguishing a particular organ from the surrounding tissue, also depends on the shape of the organ. If the shape of the object (organ) does not change significantly in the direction of the scan or along the *Z* axis, the CT numbers at the border of the organ will not be altered significantly. However, if the shape of the object varies rapidly in the *Z* axis, the CT numbers at the border region will be altered due to the partial volume effect, creating a blurred area around the border. The magnitude of the effect will depend on inter slice thickness or spatial resolution in the *Z* direction and can be reduced by selecting thinner slices. It is schematically illustrated in Fig. 5. Our data suggest that this effect becomes more evident when the accurate delineation of a given organ, depends on the difference in CT numbers on either side of its border rather than its anatomical characteristics. This could explain, in part, the absence of interscan volume difference noted for the prostate where the surrounding organs or structures are of similar densities. Another contributing factor may be the inherent variability in contouring the prostate.^8^ ^,^ ^9^

As seen on the bladder graph of Fig. 1, there seems to be a trend for smaller bladders to exhibit larger interscan volume differences. This is probably related to the shape of the bladder itself. Larger or full bladders tend to have a cylindrical shape while small bladders are more spherical. The variation of radius or diameter along the *Z* axis will be less for a cylindrical organ than a spherical one. Applying the above analysis, the observed partial volume effect will be of smaller magnitude for larger bladder because of their cylindrical shape (Fig. 2). This effect has been observed by Plewes who described that small round lesions will exhibit reduced radiographic contrast when compared to cylindrical objects of similar diameter and density.^32^ Although his study was limited to small objects, the analogy is worth noting. We suspect that this may explain in part, the absence of interscan volume difference observed for the rectum, a cylindrical organ. Other factors may include interscan variability in contouring and variations of rectal diameter and shape between the two scans.

Our analysis suggests that the differences in slice by slice areas, and hence, volumes between the 3 and 5 mm scans appear to increase when the rate of change of the bladder radius in relation to *Z*, is large as shown in Fig. 2. By analogy with the explanation given above regarding the shape of the bladder, the rate of change of the bladder radius in the *Z* direction will be larger with 5 mm slices than 3 mm slices for a given bladder. Hence, the partial volume effect will be more prominent with the 5 mm slices which in turn will lead to the averaging of CT numbers in a larger area around the border of the bladder and create a blurred region around it. Since the bladder has a clear contour (in comparison to other organs such as the rectum and the prostate), when an observer contours the bladder on each CT slice, the blurred region or area of uncertainty is excluded from the contour. This would therefore yield smaller slice by slice areas for the bladder and produce smaller bladders on the 5 mm scans as we observed (Fig. 5).

## CONCLUSIONS

In conclusion, this study has demonstrated several findings pertinent to prostate cancer radiotherapy planning:

(1) There is no trend or statistically significant association between the CT slice thickness and measurements of rectal and prostate volumes.
(2) Bladder volume is larger when 3 mm slices are used in comparison to 5 mm. This finding was demonstrated in all patients and confirmed by a second blinded observer.
(3) Partial volume effect and the resulting blurred region at the periphery of the bladder on the CT slice have been identified as the mechanism behind this interscan volume difference.
(4) The relative difference of bladder volume was ≤20% in 4/16 patients (25%) and can lead to significant differences in DVH. The clinical significance of the DVH difference, however, remains a topic for future investigations.
